# Use of non-pharmacological methods in managing labour pain: experiences of nurse-midwives in two selected district hospitals in eastern Tanzania

**DOI:** 10.1186/s12884-022-04707-x

**Published:** 2022-04-30

**Authors:** Dorkasi L. Mwakawanga, Lilian T. Mselle, Victor Z. Chikwala, Nathanael Sirili

**Affiliations:** 1 Department of Community Health Nursing, School of Nursing, Muhimbili University of Health and Allied Sciences, P.O Box 65001, Dar es Salaam, Tanzania; 2 Department of Clinical Nursing, School of Nursing, Muhimbili University of Health and Allied Sciences, P.O Box 65001, Dar es Salaam, Tanzania; 3 Department of Development Studies, School of Public Health and Social Sciences, Muhimbili University of Health and Allied Sciences, P.O Box 65454, Dar es Salaam, Tanzania

**Keywords:** Labour pain, Non-pharmacological methods, Nurse-midwives, Experiences, Tanzania

## Abstract

**Background:**

Labour pain usually brings with it many concerns for a parturient and her family. The majority of the women in labour pain may require some sort of pain relief method during this period, be it pharmacological or non-pharmacological. In Tanzania, the use of non-pharmacological methods to relief labour pain remains low among nurse-midwives. We analysed the experiences of nurse-midwives in the use of non-pharmacological methods to manage labour pain, in two selected districts of Pwani and Dar es Salaam regions in eastern Tanzania. This paper describes Non-pharmacological Methods (NPMs) currently used by nurse-midwives, the facilitators, myths and fears related to the use of NPMs.

**Materials and Methods:**

An exploratory qualitative study using in-depth interviews was conducted with 18 purposively recruited nurse-midwives working in labour wards in two selected district hospitals in Pwani and Dar es Salaam regions in eastern Tanzania. Qualitative conventional content analysis was used to generate categories describing the experience of using non-pharmacological methods in managing labour pain.

**Results:**

This study revealed that nurse-midwives encouraged women to tolerate labour pain and instructed them to change positions and to do deep breathing exercises as a means to relief labour pain. Nurse-midwives’ inner motives facilitated the use of non-pharmacological strategies for labour pain relief despite the fear of using them and myths that labour pain is necessary for childbirth.

**Conclusion:**

This study generates information about the use of non-pharmacological strategies to relief labour pain. Although nurse-midwives are motivated to apply various non-pharmacological strategies to relief labour pain, fear and misconceptions about the necessity of labour pain during childbirth prohibit the effective use of these strategies. Therefore, together with capacity building the nurse-midwives in the use of non-pharmacological strategies to relief labour pain, efforts should be made to address the misconceptions that may partly be of socio-cultural origin.

## Background

Labour pain often bring with it anxiety and a host of other concerns for a woman and her family as she approaches to labour and delivery. The labour pain depends on the woman’s earlier experience of labour pain, labour pain management, and its physical and psychological expression [1]. In some cultures, women are taught that labour pain is natural and that the ability to accept and endure labour pain is a sign of womanhood, and midwives believed there is nothing they can do about labour pain [2–4]. Consequently, cultural beliefs, values and traditions may significantly influence the experience of childbirth and attitudes towards labour pain relief [5–7]. Women`s beliefs and experience of labour pain make them anticipate intense pain, worry, fear and depression, while they shouldn’t be doing so; these aspects later influence their choice of the mode and place of delivery [7–9]. It is therefore recommended that pain relief is made a priority and an essential aspect of care for all races and cultures, satisfying women who access delivery services in healthcare facilities [10]. Many women may benefit from the midwifery model of care which emphasises the handling of labour and birth in low-risk pregnancy as a normal physiological process and promotes the incorporation of non-pharmacological methods into practice [11]. However, insufficient antenatal education and negative cultural beliefs on labour pain management have been linked with its low uptake among women and to midwives` attitude with regard to offering labour pain relief approaches [12].

Despite inadequate implementation of labour pain management, international guidelines on respectful maternity care are available to increase the quality of care, including managing pain during labour [13]. The number of women requesting pain relief medication during labour is increasing. WHO advocates labour pain management as a quality of care that should be offered to meet the highest attainable standards of health care, timely and appropriately according to the woman`s choice, culture and needs [14, 15]. Nurse-midwives working in labour wards strive to improve the quality of care through offering safe and effective labour pain relief without affecting women`s mobility or labour progress, in a woman-centered environment [4]. Labour pain can be reduced through both pharmacological and non-pharmacological interventions.

Non-pharmacological pain management (NPM) during labour refers to approaches used to increase comfort, promote rest, facilitate the woman`s coping with the labour pain, and to preventing her suffering without the use of medication [16, 17]. The NPM can be achieved through strategies like education for childbirth preparation, performing breathing exercises, frequent changes of position during labour, taking a warm bath, sitting on the birth ball, massage and listening to music [16–18]. NPM reduces labour pain with less or no effect on the mother, the foetus or the progress of labour [18, 19]. Moreover, NPM has the potential to reduce the consumption by women during labour of analgesics which may be associated with an increased risk of the delayed second stage of labour, instrumental delivery and Caesarean section [2]. The number of women accessing maternal health care is increasing [3]. In Tanzania, 98% of women attend at least one antenatal clinic and 63% of deliveries occur in a health facility, with 64% of these being assisted by a skilled provider [20]. However, the reasons for women not giving birth in a health facility are not straight forward and may include a lack of pain relief options during labour from health care providers.

Strengthening nursing and midwifery training is key for improving midwifery care [21]. Evidence shows that fully educated and regulated midwives integrated within and supported by interdisciplinary teams and an enabling environment can deliver about 90% of essential sexual, reproductive, maternal, newborn and adolescent health (SRMNAH) interventions over the life course [22]. In Tanzania, the training of nurse-midwives is organised into different levels, including certificate, diploma, bachelor, masters and PhD degrees. The certificate level is a two years` program that produces enrolled nurses who practice under the direction and delegation of a registered nurse. The diploma program is a three years training that produces a nurse-midwife who practices independently in collaboration with other healthcare professionals. A four-year bachelor degree training produces a nursing officer who works independently and supervises the provision of care, whereas a person with a two-year master's degree with advanced training is expected to provide comprehensive care according to specialties [23]. Additionally, Ph.D. training involves majoring in different areas of nursing and midwifery practice to assume various roles in academia, research, leadership and management, and in consultancy.

Effective use of NPM to relief labour pain is believed to increase satisfaction and reduce anxiety, stress and fear among women during labour which otherwise may worsen labour pain and prolong labour because of stress hormone release [19, 24]. A study done in Tanzania reported that providers are aware of various approaches to pain management including pharmacological and non-pharmacological methods however, they do not routinely apply these strategies [3]. This study analysed the experiences of nurse-midwives in Tanzania of using non-pharmacological methods in managing labour pain, as a strategy for increasing satisfaction and hence facility deliveries.

## Materials & methods

### Study design & setting

An exploratory qualitative research [25] using a semi-structured interview guide was used to explore nurse-midwives` experiences of the use of non-pharmacological methods to manage labour pain in Tanzania. The study was conducted at two district hospitals, one in Dar es Salaam region that we conveniently labelled as A and another in Pwani region that we conveniently labelled as B. Both A and B provide basic and comprehensive obstetrics and newborn care. These hospitals serve as referral hospitals for their immediate lower-level facilities. Hospital A was purposefully selected so as to represent a district hospital in a cosmopolitan area that is located in an area with a catchment of both affluent and lower socioeconomic status populations. Hospital B, although located in the urban in Pwani region serves a large majority of referrals from lower socio-economic populations. Therefore, the two facilities have relatively similar characteristics and thus could provide a better picture of NPMs use both in urban and rural areas.

### Participants and recruitment

Purposive [26] and chain referral sampling strategies were used to recruit nurse-midwives based on their years of experience. The researcher requested the nurse-midwife in charge of the labour ward in the two health facilities to provide the list of nurse-midwives working in the labour ward and involved in the direct care of women during labour and birth. The researchers grouped the participants into three groups as follows: nurse-midwives with less experience referred to nurse-midwives who had worked in the labour ward for less than one year; those with moderate experience were the ones who had worked in the labour ward for between 2 to 5 years; those with high experience had worked in the labour ward for more than 5 years. The reason for involving nurse-midwives with different years of experience was to unveil different experiences of using non-pharmacological methods for labour pain management. In each group, we approached the first participant as we were introduced by the in-charge after explaining the purpose of our visit. Each identified nurse-midwife referred us to another member having the same work experience. We recruited 10 nurse-midwives from hospital A (Dar es Salaam region) and 8 from hospital B (Pwani region). Recruitment of participants was stopped after data saturation was observed that is when there was no new information being obtained from the study participants and the redundancy had been achieved.

### Data collection

The interviews were conducted between July and November 2020. Ethical approval to conduct this study was obtained from Muhimbili University of Health and Allied Sciences (Ref. No.MUHAS-REC-2–2020-103). We collected data using a semi-structured interview guide in the Kiswahili language. The questions were constructed based on the existing literature [2, 3, 27] and the experiences of authors on labour pain management in Tanzania. Before each interview, the researchers requested written informed consent from each participant. The latter involved explaining to each study participant the purpose of the study and how the information was to be collected, including their rights to withdraw and the principles of confidentiality to each study participant. The informed consent involved consent to audio recording and publishing of the information collected.

### In-depth interviews

Eighteen (18) in-depth interviews were conducted with nurse-midwives using a semi-structured interview guide to explore experiences of nurse-midwives in the use of non-pharmacological methods to manage labour pain. A semi-structured interview guide with open-ended questions and probes was used to explore and understand better the issues of relevancy to the use of labour pain relief, as they emerged [28]. Four researchers were involved in data collection of whom two (2^nd^ and 4^th^ authors) have a considerable experience with qualitative research. Interviews were conducted in an office chosen by the participant in the buildings of the respective hospital, to ensure their comfort and privacy. Each interview was done at a time convenient for the study participant based on a pre-arranged appointment with the researcher. All interviews were conducted in the Kiswahili language as it is the native language of the participants and all the authors. Interviews were audio-recorded to capture the information provided by the participants. Field notes on verbal and non-verbal aspects were taken during the interview process to complement the audio-recorded information. We reviewed the field notes on a daily basis to improve the subsequent interviews and to note the emerging findings. It was not until we began the 11^th^ interview that we realised the recurrence of similar issues, with few new findings. At the 18^th^ interview, we decided to stop further interviews as there was no new information emerging anymore. We, therefore, considered the 18^th^ interview as our point of information saturation [29]. Each interview lasted between 30 to 60 min.

### Data analysis

Qualitative content analysis following Graneheim and Lundman [30] guided the analysis of data. Audio-recorded interviews were first transcribed verbatim. We analysed the transcripts in the Kiswahili language to maintain the originality of the gathered information, as all the coders were fluent and native Kiswahili speakers. The analysis was conducted by four researchers DLM, LTM, VZC and NS, who worked in pairs to ensure reliability [31]. The complete transcripts and field notes were first read and re-read by all authors to become familiar with the data and the context. The qualitative data analysis NVivo software was used in managing and organising data. Condensed meaning units related to participants` experiences of using non-pharmacological labour pain relief measures were formed through data reduction. The codes were then generated from condensed meaning units. The initial list of codes was then discussed by all authors and the agreement was reached on the final codes. Similar codes were grouped and abstracted into sub-categories through comparison. Through checking on similarities and differences of sub-categories and reflection upon the interpretations of the participants` experience descriptions, the authors discussed and agreed on the main categories. Then succinct quotes were selected to support the categories and sub-categories. Finally, the codes and quotes were translated from Kiswahili to English and presented to illustrate the facts using the participants` accounts. Although the description seems to be linear, the process of analysis was iterative.

### Methodological considerations

To ensure the trustworthiness of this study, we used four Lincoln and Guba criteria of credibility, dependability, transferability and conformability [32]. Credibility was ensured by collecting data from two different hospitals (rural and urban) to ensure comprehensive accounts of the experiences of nurse-midwives [25, 33]. Dependability was ensured by doing initial analysis by all authors and thereafter agreeing on the codes. Validation of data accuracy was achieved through a member check [34]. Participants’ quotes have been included to support the researchers’ interpretations, which also increases the credibility of the study [35]. Dependability and confirmability were promoted through an inquiry audit, whereby the researchers reviewed and examined the research process and the data analysis to ensure that the findings were consistent. Description of the study design and setting, the purposive sampling approach used, the data collection method that was used, the description of the analysis process and the use of participants` accounts will allow determining the study`s transferability to other contexts [36].

## Results

We interviewed 18 nurse-midwives about their experiences of the use of non-pharmacological methods in managing labour pain. The age range of the participants was between 25 and 53 years. All participants were females. Four had worked in a labour ward for less than one year, six had worked for between 2 to 5 years and eight had more than 5 years` experience of working in the labour ward. Their education level ranged from certificate to bachelor`s degree, with six having a certificate, ten having a diploma and two having a bachelor`s degree.

The analysis of data identified three major categories that describe the nurse-midwives experiences with the use of non-pharmacological methods in managing labour pain. These included the non-pharmacological approaches used by nurse-midwives, facilitators for using non-pharmacological methods and the myths and fears regarding the use of non-pharmacological methods (Table 1).

**Table 1:** Summary of findings

Selected codes	Sub-categories	Categories
• Telling women to change lying positions • Instructions to women to open mouths and take a deep breath • Encourage movements like walking	Encouragements and instructions on exercises, position changing and deep mouth breathing	Non-pharmacological approaches used by nurse-midwives in managing labour pain
• Talking to women about labour prepares their mind • Telling women are going to deliver safely • Telling women to tolerate pain	Providing psychological support	
• Rubbing women back • Massaging women`s backs when not busy • Massaging a woman`s back when in contraction	Performing a back massage	
• Doing what one`s love doing • Motivation from my heart • The desire comes from my inner heart	Nurse-midwives intrinsic motivation	Facilitators for using non-pharmacological methods in managing labour pain
• Knowing the NPMs • Women appreciations • Experience in using the methods	The comfort of nurse-midwives on using the methods	
• NPMs relief pain • Raise women`s confidence • Reduces incidence of fetal distress	Understanding the usefulness and benefits of using NPMs	
• The absence of labour is a sign of poor progress • Relieving pain cause a delay to give birth • Pain is viewed as necessary for a baby to come out	Misconceptions about labour pain relief	Myths and fears related to using non-pharmacological strategies to relieve labour pains
• Women feel disturbed • Women do not accept • Women never follow our instructions	Women may be uncomfortable with the methods	
• The baby may fall • The baby may fall on the floor	Fear of baby falling	

### Non-pharmacological approaches used in managing labour pain

Non-Pharmacological approaches as revealed included encouragement and instructions provision of psychological support and back massage.

Nurse-midwives instructed labouring women to perform exercises including changing positions and conducting deep mouth breathing exercises between contractions. They further encouraged women to walk around the bed and at times to squat when the pain became intense, to relieve pain. A nurse-midwife reported;

*“When a woman is in pain, I tell her to take a deep breath, which will help her not to get tired and the pain to be less intense. So, I teach women how to perform mouth breathing. I have seen this being so helpful”. (NM1, 1-year experience)*

Providing psychological support was another NPM stated by nurse-midwives as being used to relieve labour pain. Nurse-midwives added that at the onset they informed women about labour pain and what they should expect, and they believe that this information prepared the women psychologically to deal with labour pain.

*”I always let the mother understand that, as time goes there will be a series of events concerning her pain. This prepares her mind. I tell the woman to tolerate and endure the pain, which will make her feel less pain, (…) the reassurance about safe delivery prepares her psychologically as well”. (NM10, 2 years experience)*

Back massage was another NPM approach used by the nurse-midwives to relieve their clients` labour pain. Participants acknowledged that rubbing the lower back of the woman in labour provides immediate pain relief.

*“…another method I sometimes use is massaging a woman on her back, which I know reduces pain instantly, but we don’t use it often as we (nurse-midwives) are fewer in number than the clients we get; but I know it helps to reduce labour pain”. (NM13, 4 years` experience)*

### Facilitators for using non-pharmacological methods in managing labour pain

Nurse-midwives` intrinsic motivation, their comfort with using the methods, as well as their understanding of the usefulness and benefits of using NPMs were stated to facilitate the use of NPM in managing labour pain.

Intrinsic motivation was stated by nurse-midwives to promote the use of NPM to manage labour pain. Furthermore, nurse-midwives stated that the shortage of nurse-midwives acts as a barrier to the use of NPM.

*“It comes from my heart, the spirit tells me to do something I like, you know it feels good when you do something you love, people do say becoming a midwife must come from your heart`. I mean, this is something that developed from your heart, and you will never wait for someone to tell you to do something to help mothers”. (NM 11, 15 years` experience)*

Nurse-midwives stated that their comfort with using the methods was another facilitator towards using NPM in managing labour pain. They added that being knowledgeable and skilled and having experience in using the methods promote the nurse-midwives` use of NPM in managing labour pain.

*“Knowledge and skills also inspire us to use, as I can comfortably apply what I learned or experienced, like performing a back massage, telling the mother to exercise regularly, because I know it's a good thing to be done and is helpful”. (NM5, 10 years` experience)*

Understanding the usefulness and benefits of using non-pharmacological methods in managing labour pain has also emerged from the participants` descriptions. Participants stated that the use of labour pain relief measures not only reduces the pain, but also promotes close provider–client close relationships and increases the mother`s comfort and confidence during the childbirth process.

*“…it builds a close relationship with a woman, she becomes more comfortable and confident, she feels to be at the right place. She learns that performing breathing exercises, changing position will help to relieve her pain when contractions are too strong”. (NM1, 1-years` experience)*

### Myths and fears related to using non-pharmacological strategies to relieve labour pain

Misconceptions about labour pain relief, women being uncomfortable with the methods, and fear of the baby falling emerged as myths and fears attributed to the use of NPM in managing labour pain. Some midwives reported not using NPM to relieve labour pain, believing that childbirth should be painful. They further added that they thought the absence of pain may be an indication of poor progress of labour. These misconceptions of viewing pain as necessary during labour made them fear to use pain relief measures.

*“The feeling of pain is beneficial to the woman in the way that if she doesn’t feel pain means she is not progressing well (….) The presence of pain gives reassurance of the foetus descending thus if the pain subsides means the delivery may also be difficult”. (NM14, 3-years` experience)*

Participants reported fear of using pain relief measures due to their experience of how women felt about the pain relief measures used. Participants stated that, regardless of the few available labour pain relief measures they tried to use, some women refused, felt disturbed by providers and did not follow the instructions given by providers which led to the concern that women do not feel comfortable with the methods.

*“Some women refuse the pain relief measures to be performed, they find it annoying and do not accept, complain to feel bad, so it needs extra effort to make them understand”. (NM7, 5 years` experience)*

Nurse-midwives reported a fear of the baby falling following the use of some pain relief measures such as exercising and position changing. Participants stated that, although this may not happen often, some women assume that exercise such as walking, with uncertainty about the level of the foetus may pose a risk of the baby falling on the floor.

“*The effect of the baby falling on the floor…. however very rare can occur when a woman performs movements like walking without knowing the level of the foetus (……); as a provider, I may tell a woman to get out of bed and exercise without knowing the baby is so close”. (NM5, 8 years` experience)*

## Discussion

We explored the experiences regarding the use of non-pharmacological methods in managing labour pain in Tanzania, based on the perspectives of nurse-midwives. Our study revealed the common non-pharmacological approaches used by nurse-midwives in managing labour pain, facilitators for using non-pharmacological methods in managing labour pain, and the myths and fears regarding the use of non-pharmacological strategies to relieve labour pain.

Although the topic was new and of surprise to most of the participants, many of them reported using several non-pharmacological methods in managing labour pain. These include the provision of psychological support, back massage, encouragements and giving instructions to mothers on breathing techniques (deep mouth breathing), position change during labour and exercising. This is similar to what was found in other studies, where the majority of midwives reported using various non-pharmacological methods, including changing a woman`s position which encourages labour progress and increases cervical dilatation [3, 10, 37]. Recent studies recommend labour pain relief for higher maternal satisfaction with childbirth and reduction of obstetric interventions including the Caesarean section [10, 24, 38]. However, other non-pharmacological methods such as education for childbirth preparation, warm bath/shower and music which can be effectively used in our context were not reported by any of the participants, signifying limited awareness or rare use of these methods among our participants. This is not unique to Tanzania; similar findings have been reported in Iran, Ghana and Ethiopia, where many methods of non-pharmacological pain relief are not well known to the majority of health care providers, who are thus unable to offer non-pharmacological methods to manage labour pain [2, 10, 27, 37]. This shows that the use of NPMs by health care providers is still low and the management of labour pain is not evidence-based. This is because the methods for pain relief are not emphasised in the nursing and midwifery training and therefore nurse-midwives lack in using them [2, 39].

Oral fluid and food intake in labour has been encouraged to enhance energy and stamina and its restriction has no beneficial effects on important clinical outcomes [40, 41]. In this study, it was noted that nurse-midwives encouraged women to take fluids such as hot tea and porridge during labour because they consider it may ease the pain. Moreover, psychological support was strongly noted to be the most common approach used in managing labour pain by the majority of participants. This is done through counselling women on labour pain, providing reassurance, good care, attention, support and consolation to mothers in labour. Also, nurse-midwives provided information on the labour process and progression after the assessment as one way of making women relax and cope with pain when they didn’t have enough time to use other approaches such as sacral massage which is believed to be more efficient in pain relief. This is strongly backed by other studies which reported that continuous support during labour including psychological support was closely related to less pain and a satisfying childbirth experience [42, 43].

The intrinsic motivation among nurse-midwives has been among the facilitators for using non-pharmacological methods in managing labour pain. This has been characterised by their passion and inspiration to help women and the desire to use such approaches to help mothers during labour. The participants recognised that knowledge of non-pharmacological methods used in managing labour pain increases nurse-midwives` comfort and confidence in using the various approaches. Our study revealed that the use of non-pharmacological methods in managing labour pain was also facilitated by the experience of having gone through labour pain. This finding is in line with what was found in Ghana that a midwife who has gone through labour pain is more likely to demonstrate a higher estimation of labour pain and could have empathy for labouring women [37]. The possible reason for this comparison may be due to similarities in culture and context.

In the current findings, the nurse-midwives understood the usefulness and benefits of using non-pharmacological methods in managing labour pain and hence their desire to use them. As revealed in our study, similarly, increasing women`s comfort and self-confidence and their being able to cope with pain were the most reported benefits [2, 12, 44]. Moreover, the increase in hospital deliveries due to a positive childbirth experience was found to be the important aspect as stated by the majority. This was reported to be an outcome once women get satisfied with the care provided in health facilities during labour and delivery. Despite the good progress, the health facility deliveries in Tanzania are still low [45, 46], and the possible reason may be the negative childbirth experiences that reinforce mistrust of health facility care. As recommended by WHO and SDGs the standard for quality of care provided at a health facility in increasing women's satisfaction includes timely and appropriate pain management offered to women during labour to facilitate a humanised childbirth experience [1, 14, 15, 24]. There is now a window of opportunity to adapt and amend the available labour wards` protocols/guidelines to include the provision of non-pharmacological labour pain relief measures as an integral component of quality of care, so as to increase hospital deliveries.

The myths regarding the use of labour pain relief approaches reported in this study were associated with misconceptions and beliefs about labour and labour pain management. Some of the nurse-midwives believed that without pain a baby could not come out and that reducing pain may lead to a labour prolongation. In line with other findings, they believed that pain is necessary for a woman in labour and that its absence may be a sign of a problem in labour progression [2–4]. This is an indication of a knowledge gap in labour pain management, as to how socio-cultural beliefs may influence health care practices. This calls for the debate on how to address the socio-cultural issues in training as well as provide orientation to health care providers on labour pain management options to empower them with knowledge, thereby facilitating their use. The findings also revealed that nurse-midwives feared that there is a possibility of the baby falling in case some approaches are used without assessing the level of the baby. Although this sounds like a non-objective, it is important to take note of this, as it provides a basis regarding the knowledge gap on the labour pain management options based on the labour phases.

## Conclusion

Nurse-midwives use various non-pharmacological methods to relieve labour pain. These include encouraging women and providing instructions on changing positions, using deep mouth breathing techniques and exercises, providing psychological support and performing sacral massage. However, these strategies and several other approaches were not commonly used because the majority were unfamiliar with the methods and how they are used. Intrinsic motivation and passion were key drivers for using non-pharmacological labour pain approaches. Nevertheless, nurse-midwives reported some myths and fears that prevented the optimal use of labour pain management approaches. This indicates the knowledge gap, which shows the need for designing an intervention to fill the gap, including comprehensive labour pain management education in health professionals` training programs in Tanzania. To move forward with the provision of standard quality care for pregnant women during labour and delivery, the emphasis should be on labour pain management as a component of humanising childbirth, thereby raising the number of health facility deliveries. We recommend further studies to explore the perspectives of the significant others and of the community in general on labour pain management, considering their key role in preparing pregnant women for labour and birth.

## Data Availability

The data analysed during the current study are available from the corresponding author upon request.

## Abbreviations

MUHAS: Muhimbili University of Health and Allied Sciences
NM: Nurse-midwife
NPM: Non-pharmacological Pain Management
SDG: Sustainable Development Goals
SIDA: Swedish International Development Cooperation Agency
SRMNAH: Sexual, Reproductive, Maternal, Newborn and Adolescent Health
WHO: World Health Organizations
